# A Role for Autophagy in the Extension of Lifespan by Dietary Restriction in C. elegans

**DOI:** 10.1371/journal.pgen.0040024

**Published:** 2008-02-15

**Authors:** Malene Hansen, Abha Chandra, Laura L Mitic, Brian Onken, Monica Driscoll, Cynthia Kenyon

**Affiliations:** 1 Department of Biochemistry and Biophysics, University of California San Francisco, San Francisco, California, United States of America; 2 Department of Molecular Biology and Biochemistry, Rutgers University, Piscataway, New Jersey, United States of America; Stanford University Medical Center, United States of America

## Abstract

In many organisms, dietary restriction appears to extend lifespan, at least in part, by down-regulating the nutrient-sensor TOR (Target Of Rapamycin). TOR inhibition elicits autophagy, the large-scale recycling of cytoplasmic macromolecules and organelles. In this study, we asked whether autophagy might contribute to the lifespan extension induced by dietary restriction in C. elegans. We find that dietary restriction and TOR inhibition produce an autophagic phenotype and that inhibiting genes required for autophagy prevents dietary restriction and TOR inhibition from extending lifespan. The longevity response to dietary restriction in C. elegans requires the PHA-4 transcription factor. We find that the autophagic response to dietary restriction also requires PHA-4 activity, indicating that autophagy is a transcriptionally regulated response to food limitation. In spite of the rejuvenating effect that autophagy is predicted to have on cells, our findings suggest that autophagy is not sufficient to extend lifespan. Long-lived *daf-2* insulin/IGF-1 receptor mutants require both autophagy and the transcription factor DAF-16/FOXO for their longevity, but we find that autophagy takes place in the absence of DAF-16. Perhaps autophagy is not sufficient for lifespan extension because although it provides raw material for new macromolecular synthesis, DAF-16/FOXO must program the cells to recycle this raw material into cell-protective longevity proteins.

## Author Summary

Dietary restriction (limited food intake) increases lifespan in many organisms. However, the cellular processes underlying this fascinating phenomenon are still poorly understood. When an animal is starved, it degrades and recycles its organelles and other cellular components in a process called autophagy (literally “self-eating”). Here, we have asked whether autophagy also occurs in response to dietary restriction, using the roundworm C. elegans for our studies. We find that autophagy does take place when food intake is limited. Moreover, inhibiting genes required for autophagy has little effect on well-fed animals but prevents food limitation from extending lifespan. This autophagy requires PHA-4/FOXA, a life-extension protein that regulates gene expression, suggesting that changes in gene expression are required for dietary restriction to stimulate autophagy. Because autophagy seems like such a rejuvenating process, it might seem to be sufficient to increase longevity. However, we find that, in long-lived hormone-pathway mutants, both autophagy and DAF-16/FOXO, another protein that controls gene expression, are required for longevity. We propose that autophagy frees up new resources for the cell, but that transcription factors like the DAF-16/FOXO protein must channel this raw material into new cell-protective proteins in order for lifespan to be increased.

## Introduction

Dietary restriction, the reduced intake of food without malnutrition, increases the lifespan of many organisms, from yeast to mammals [1]. Dietary restriction increases lifespan, at least in part, by reducing the activities of pathways involved in growth and nutrient processing, including the TOR (Target Of Rapamycin) pathway. Inhibition of the TOR pathway extends lifespan in yeast, worms and flies [2–5], and dietary restriction cannot further extend the lifespans of yeast, worms or flies in which the TOR pathway has been inhibited [3,4,6]. This suggests that down-regulation of the TOR pathway plays an important role in the longevity response to food limitation.

TOR regulates several processes that could be involved in the longevity response to dietary restriction. For instance, TOR stimulates protein synthesis in yeast and in mammals by modulating key components of the translation machinery, including the ribosomal-protein S6 kinase (S6K) and the translation initiation factor 4E-binding protein (4E-BP). Inhibition of positive regulators of translation, including S6K, extends lifespan in both worms and flies [3, 6–10] and inhibition of the negative regulator 4E-BP shortens lifespan in flies [11]. One could imagine that TOR inhibition extends lifespan solely by inhibiting protein synthesis. However, another process regulated by TOR, autophagy [12], could also potentially influence the longevity of animals subjected to dietary restriction.

Macroautophagy (hereafter referred to as autophagy) is a process in which portions of the cytoplasm, including mitochondria and other organelles, are degraded under conditions of nutrient limitation, allowing cellular macromolecules to be catabolized and recycled. During autophagy, large double-membrane vesicles, called autophagosomes, are generated and degraded in lysosomes, together with their contents. The breakdown products are subsequently recycled to the cytoplasm [13]. The regulation of autophagy has been studied extensively in yeast [14]. In this organism, autophagy is controlled by the *ATG* genes, many of which have functional homologs in other organisms [13,15]. In yeast, TOR inhibits the protein kinase Atg1, which would otherwise mediate an early activation step in the autophagic process [16]. In response to Atg1 activity, the Vps34 complex, which contains the Class III phosphatidylinositol-3-kinase Vps34 as well as Atg6/Vps30, the ortholog of the mammalian protein Beclin1, stimulates and nucleates the formation of autophagosomes [14,17,18].

Autophagy is induced under conditions of stress, including nutrient limitation. For instance, dietary restriction stimulates autophagy in old rodents [19–21], and in C. elegans larvae that enter a state of diapause, called dauer, in response to food limitation and crowding [22]. The process of autophagy has been linked to lifespan extension in long-lived insulin/IGF-1-pathway mutants. Mutations in components of the insulin/IGF-1 signaling pathway extend lifespan in many organisms [23,24]. In C. elegans, strong inhibition of the insulin/IGF-1 signaling pathway induces dauer formation, and weaker inhibition permits growth to adulthood and extends adult lifespan. Both *daf-2-*mutant dauers and adults exhibit increased levels of autophagy, and autophagy is required for their long adult lifespans [22]. RNAi inhibition of several autophagy genes, including *ATG6/beclin1/bec-1*, prevents *daf-2* mutations from extending lifespan, but has only minor effects on the lifespan of wild-type animals [22,25].

Despite the link between nutrient limitation and autophagy, it is not known whether autophagy plays a direct role in the longevity response to dietary restriction. In this study, we find that both dietary restriction and inhibition of the TOR pathway stimulate autophagy in *C. elegans,* and inactivation of genes required for autophagy specifically prevents these conditions from extending lifespan. We find that autophagy, like lifespan extension itself, is not a passive consequence of food limitation, but instead involves specific transcriptional control. Finally, our findings indicate that autophagy is neither necessary nor sufficient to extend lifespan in *C. elegans,* rather, autophagy appears to be an essential aspect of certain longevity pathways that are linked to nutrition.

## Results

### Dietary Restriction Triggers Autophagy in C. elegans

To address the role of autophagy in the longevity response to dietary restriction, we made use of *eat-2(ad1116)* mutants, which are a genetic model for dietary restriction in C. elegans [26]. These mutants have defects in a pharyngeal nicotinic acetylcholine receptor subunit that is required for pharyngeal pumping (feeding) [27]. *eat-2* mutants are long lived, and share many characteristics of animals that are directly food limited. These include a pale, thin morphology [26], a lifespan extension that is dependent on the *pha-4*/FOXA transcription factor [28], but independent of *daf-16*/FOXO (a transcription factor required for the longevity of *daf-2* insulin/IGF-1-receptor mutants) [26,29], reduced and prolonged progeny production [30], and a characteristic spectrofluorimetric profile [31].

**Figure 1:**
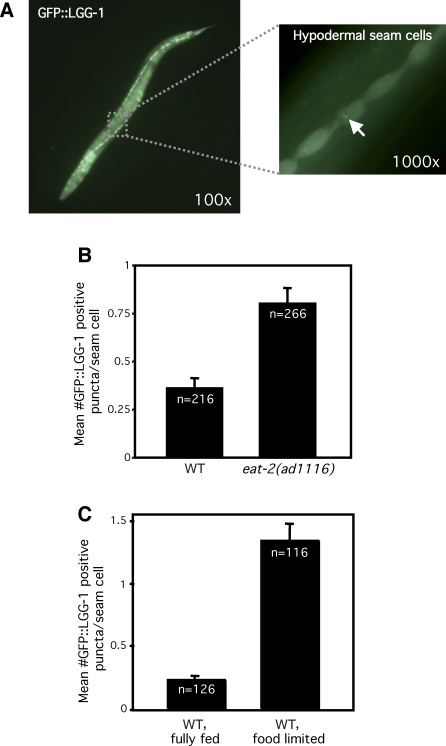
Dietary Restriction Increases the Level of Autophagy LGG-1::GFP-positive puncta labeling autophagic membranes [22] were counted in wild-type or in food-limited animals. (A) Micrographs of *eat-2(ad1116)* L3 larvae expressing GFP-tagged *lgg-1*/LC3. Arrow indicates autophagic focus. Magnification is indicated. (B) Average number of LGG-1::GFP-containing puncta in *eat-2(ad1116)* mutants and N2 wild-type animals (WT), *p* < 0.0001. (C) Average number of LGG-1::GFP-containing puncta in N2 wild-type, food-restricted animals grown in liquid media (WT, food limited) and N2 wild-type animals grown in liquid with a higher concentration of bacteria (WT, fully fed), *p* < 0.0001; see Methods. Between three and ten seam cells were counted in each of 20–40 animals using high-power microscopy and averaged. *n*, total number of seam cells observed. Error bars: ±SEM. *p*-Values were calculated as unpaired, two-tailed t-test. Animals were raised at 20 °C. Please see Table S1 for quantification of all data.

While these studies were in progress, Pilon's group reported increased levels of autophagy during the development of several feeding defective C. elegans mutants whose adult longevity phenotypes have not been well characterized [32]. To ask if autophagy occurs in *eat-2* mutants and in animals subjected to direct dietary restriction, we visualized a GFP-tagged LGG-1 protein involved in autophagy (Figure 1A) [22]. LGG-1 is the worm ortholog of the vacuolar protein Atg8/MAP-LC3, which is incorporated into pre-autophagosomal and autophagosomal membranes. In C. elegans, LGG-1::GFP is localized to puncta or foci in cells that are known to have increased numbers of autophagic vesicles [22,33]. The appearance of LGG-1::GFP-containing puncta has been used widely as an indicator of autophagy in C. elegans [22,32–35]. We found that the low number of autophagic events in wild-type L3 animals was increased ∼2.5-fold in *eat-2(ad1116)* mutants (Figure 1B, *p* < 0.0001, t-test). The longevity response to dietary restriction can be triggered in adults, and, consistent with this, we also observed increased levels of LGG-1::GFP-containing foci in the seam cells of adult *eat-2* animals (data not shown). In addition, we found that wild-type L3 animals subjected to direct dietary restriction by food limitation [30,36] exhibited a large increase in the number of autophagic puncta (Figure 1C).

### The Autophagy-Associated Genes *bec-1* and *vps-34* Are Required for the Longevity of *eat-2* Mutants

**Figure 2:**
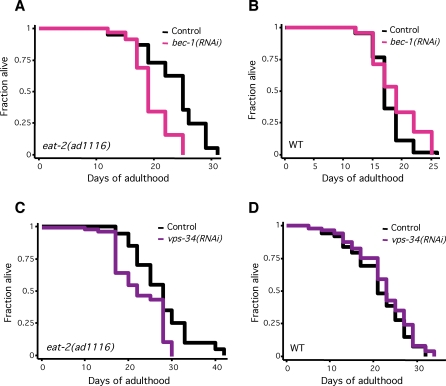
Inhibition of Genes Required for Autophagy Shortens the Long Lifespan of *eat-2* Mutants (A) Survival curves of *eat-2(ad1116)* animals fed either control bacteria or bacteria expressing *bec-1* dsRNA during adulthood at 20 °C. Mean lifespan was 23.7 d for control and 19.6 d for *bec-1* RNAi*, p* < 0.0001, Log-rank (Mantel-Cox) test. This experiment was performed a total of six times, and *bec-1* RNAi shortened the lifespan of *eat-2* animals ∼15%–30%. Please see Table 1 for additional data. (B) Survival curves of N2 wild-type animals (WT) fed either control bacteria or bacteria expressing *bec-1* dsRNA during adulthood at 20 °C. These assays were performed concurrently with the *eat-2* mutant lifespan analysis in Figure 2A. Mean lifespan was 17.3 d for control and 18.9 d for *bec-1* RNAi. *p* = 0.045, Log-rank (Mantel-Cox) test. Depletion of *bec-1* did not significantly change the lifespan of N2 or sterile *fer-15(b26); fem-1(hc17)* animals in any of six experiments. Please see Table 1 for additional data. (C) Survival curves of *eat-2(ad1116)* animals fed either control bacteria or bacteria expressing *vps-34* dsRNA during adulthood at 20 °C. Mean lifespan was 27.6 d for control and 22.8 d for *vps-34* RNAi*, p* = 0.0003, Log-rank (Mantel-Cox) test. This experiment was performed a total of four times. Please see Table 1 for additional data. (D) Survival curves of sterile *fer-15(b26); fem-1(hc17)* animals (WT) fed either control bacteria or bacteria expressing *vps-34* dsRNA during adulthood at 20 °C. These assays were performed at the same time as the *eat-2* lifespan analysis shown in Figure 2C. Mean lifespan was 21.5 d for control and 23.3 d for *bec-1* RNAi. *p* = 0.14, Log-rank (Mantel-Cox) test. Depletion of *vps-34* did not significantly change the lifespan of N2 or sterile *fer-15(b26); fem-1(hc17)* animals in each of six different experiments. Please see Table 1 for additional data.

Is autophagy required for the long lifespan induced by dietary restriction? To investigate this, we inhibited the autophagic gene *ATG6*/*beclin1*/*bec-1* in *eat-2* mutants using RNAi. Because dietary restriction extends lifespan when initiated during adulthood [36], we subjected the animals to RNAi on day-1 of adulthood by transferring them to culture dishes containing bacteria expressing *bec-1* dsRNA. In this way, we were able to circumvent the requirement for *bec-1* function during development [37]. We found that both of two different *bec-1* RNAi clones shortened the mean lifespan of *eat-2(ad1116)* mutants by ∼15–30% (Figure 2A; Table 1), but did not shorten wild-type lifespan (Figure 2B; Table 1).

**Table 1:**
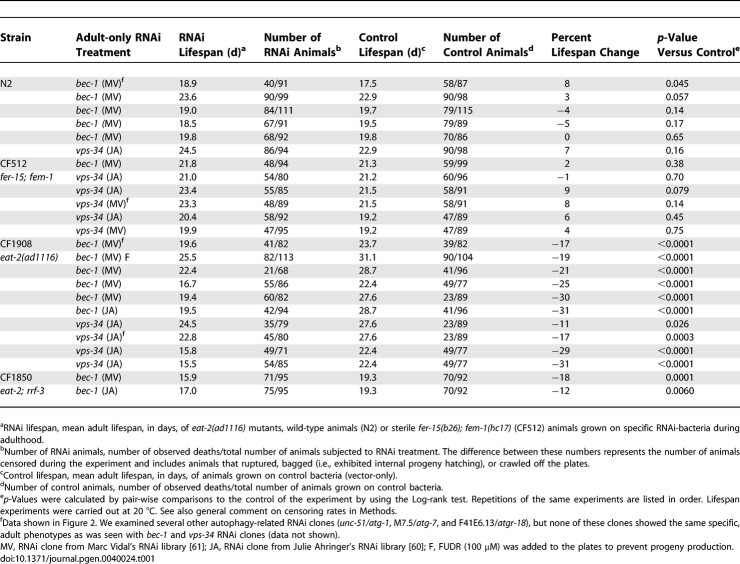
Lifespan Analysis of *eat-2* Mutants Subjected to *bec-1* or *vps-34* RNAi during Adulthood

**Figure 3:**
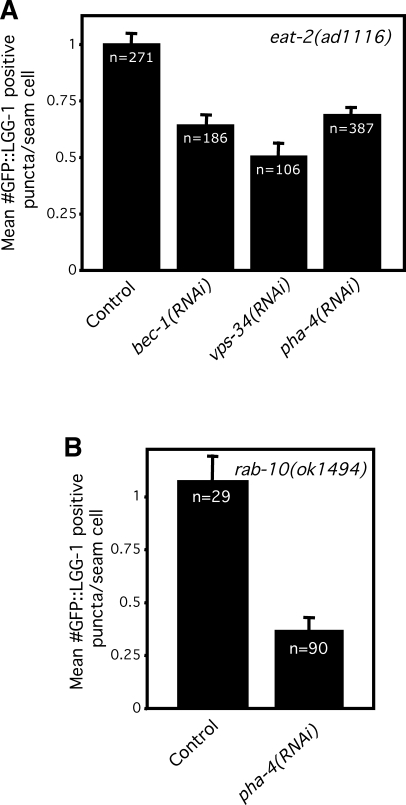
Inhibition of the FOXA Transcription Factor *pha-4* Decreases Autophagy in *eat-2* and *rab-10* Mutants (A) Average number of LGG-1::GFP-containing puncta in *eat-2(ad1116)* progeny of animals fed either control bacteria or bacteria expressing *bec-1*, *vps-34* or *pha-4* dsRNA their entire lives. *p* ≤ 0.0001 for *bec-1, vps-34* and *pha-4* RNAi treatments compared to control, respectively, unpaired, two-tailed t-test. *n*, total number of seam cells observed. Error bars: ±SEM. See Figure 1 for details. (B) Average number of LGG-1::GFP-containing puncta in *rab-10(ok1494)* progeny of animals fed either control bacteria or bacteria expressing *pha-4* dsRNA their entire lives; *p* < 0.0001, unpaired, two-tailed t-test. *n*, total number of seam cells observed. Error bars: ±SEM. Please see Figure 1 for details and Table S1 for quantification of data. Feeding mutants for several generations with *pha-4* dsRNA, inhibited development and sharply decreased the number of eggs laid ([46], data not shown). However, the loss of many embryos was unlikely to bias our findings, since many progeny of *daf-2* mutants treated with *pha-4* RNAi also died but those that did not exhibited a robust autophagy phenotype (Figure 6).

In C. elegans, BEC-1 interacts with the class III PI3 kinase VPS-34 (LET-512) [37], an essential protein required for autophagy, membrane trafficking and endocytosis. We therefore asked whether *vps-34* was also required for the long lifespan of *eat-2* mutants. As with *bec-1* RNAi, treating *eat-2* mutants with *vps-34* RNAi on day-1 of adulthood significantly shortened their long lifespan, but not that of wild type (Figure 2C and 2D; Table 1). Consistent with a role for *bec-1* and *vps-34* in autophagy, *bec-1* and *vps-34* RNAi disturbed the morphology and reduced the number of LGG-1 foci in the L3 progeny of *eat-2* animals exposed to RNAi for their entire life (see Methods) (Figure 3A and data not shown). We looked for a similar perturbation in LGG-1::GFP puncta under the RNAi conditions that we used to assay lifespan; that is, in *eat-2* mutants treated with *bec-1* or *vps-34* RNAi from day-1 of adulthood. We did not observe a change in the LGG-1::GFP pattern within the first two days of adulthood (in this or any other adult-only RNAi treatment we performed, including our experiments with *daf-2* mutants [data not shown]). After day-2 of adulthood, the level of endogenous fluorescence, which increases with age, overwhelmed the LGG-1::GFP signal (see Methods). Thus later disruption of the LGG-1::GFP pattern, which seems likely, could not be observed.

BEC-1/Beclin1 is also known to interact with CED-9/Bcl-2 [37,38], a protein that inhibits apoptosis. Therefore, we repeated the *bec-1-*RNAi experiment in animals in which cell death had been prevented using a caspase mutation, *ced-3(n1289)*. We found that *bec-1* RNAi shortened the lifespan of *ced-3(-); eat-2(-)* mutants, as with *eat-2* *(-)* single mutants (data not shown), arguing against a longevity role for *bec-1* in apoptosis. Taken together, these findings imply a requirement for autophagy in the longevity response to dietary restriction.

Next, we asked whether RNAi treatments predicted to disrupt autophagy affected other phenotypes produced by dietary restriction. We found that *eat-2(ad1116)* mutants fed *bec-1* or *vps-34* RNAi-bacteria from hatching had the same low pumping rates as *eat-2(ad1116)* animals raised on control bacteria (Figure S1). In addition, feeding *bec-1* RNAi-bacteria to *eat-2(ad1116)* animals did not have any effect on the brood size or the timing of the progeny production (Figure S2). We also asked whether inhibition of *bec-1* affected the characteristic spectrophotometric spectrum of *eat-2-*mutant adults. Aging worms normally accumulate various fluorescent compounds that have a distinctive absorption maximum, and *eat-2* mutants and wild-type animals subjected to dietary restriction exhibit a decrease in the absorption maximum of these age-related pigments [31]. We found that *bec-1* RNAi fed to animals during adulthood did not significantly alter the fluorimetric profile of *eat-2* mutants (Figure S3). Together these findings suggest that autophagy is specifically required for the longevity response to dietary restriction.

### Mutants with Low TOR Pathway Activity Have Increased Autophagy and Require *bec-1* to Live Long

**Figure 4:**
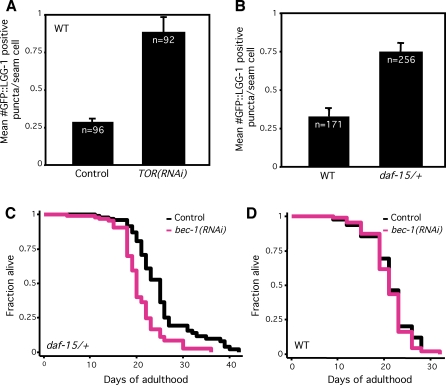
Animals with Low TOR-Pathway Activity Exhibit Increased Autophagy and Require the Autophagy-associated Gene *bec-1* during Adulthood to Live Long (A) Average number of LGG-1::GFP-containing puncta in *let-363*/TOR RNAi-arrested animals compared to N2 wild-type animals (WT) grown on control (vector-only) bacteria, *p* < 0.0001, unpaired, two-tailed t-test. *n*, total number of seam cells observed. Error bars: ±SEM. See Figure 1 for details. We were not able to detect increased LGG-1::GFP puncta in long-lived *let-363(RNAi)* adults; however, one generation of *daf-2* RNAi, our positive control, did not significantly increase the number of foci in adults either (data not shown). Please see Table S1 for quantification of all data. (B) Average number of LGG-1::GFP-containing puncta in *daf-15(m81)/unc-24(e138)* heterozygotes (*daf-15/+)* compared to N2 wild-type animals (WT), *p* < 0.0001, unpaired, two-tailed t-test. *n*, total number of seam cells observed. Error bars: ±SEM. See Figure 1 for details. Please see Table S1 for quantification of all data. *daf-15* encodes the TOR-binding partner Raptor. (C) Survival curves of *daf-15(m81)/unc-24(e138)* heterozygotes (*daf-15/+,* strain DR412) fed either control bacteria or bacteria expressing *bec-1* dsRNA during adulthood at 20 °C. Mean lifespan: *daf-15/+* animals grown on control RNAi-bacteria: 25.1 d, *daf-15/+*animals on *bec-1* RNAi: 20.8 d, *p* = 0.0008, Log-rank (Mantel-Cox) test. The lifespan of *daf-15/+* animals grown on *bec-1* RNAi-bacteria during adulthood was measured again, yielding similar results; the lifespan of *daf-15/+* animals was also measured three times following whole-life RNAi exposure. In these experiments, *bec-1* RNAi generally shortened the mean lifespan of *daf-15/+* animals to a greater extent than it shortened the lifespan of wild type. (We also attempted to perform double-RNAi experiments, in which animals were cultured on a 50:50 mixture of *let-363/*TOR and *bec-1* [or control] RNAi bacteria. Although the trends we saw were in the expected direction, the effects produced by half-strength RNAi were small and not statistically significant [data not shown].) Please see Table 2 for additional data. (D) Survival curves of wild-type animals derived from strain DR412 (WT) fed either control bacteria or bacteria expressing *bec-1* dsRNA throughout their whole life at 20 °C. These assays were performed concurrently with the *daf-15/+* lifespan analysis shown in Figure 4C. WT grown on control RNAi-bacteria: 21.4 d, WT on *bec-1* RNAi-bacteria: 21.1 d (*p* = 0.34), *p* between *daf-15/+* and WT grown on control RNAi-bacteria, *p* < 0.0001, Log-rank (Mantel-Cox) test. Please see Table 2 for additional data.

How might dietary restriction induce autophagy? As described above, dietary restriction appears to extend lifespan, at least in part, by down-regulating the TOR pathway, and inhibition of TOR is known to trigger autophagy in yeast and mammals [12]. To ask whether this was also the case in C. elegans, we assayed the levels of LGG-1 puncta in animals fed bacteria expressing TOR (*let-363*) dsRNA. When we fed wild-type animals TOR RNAi-bacteria for their entire lives, we observed a significant increase in the number of autophagic vesicles in their L3 progeny (Figure 4A), whose development, like that of *let-363(h98)/*TOR mutants, was arrested [2].

**Table 2:**
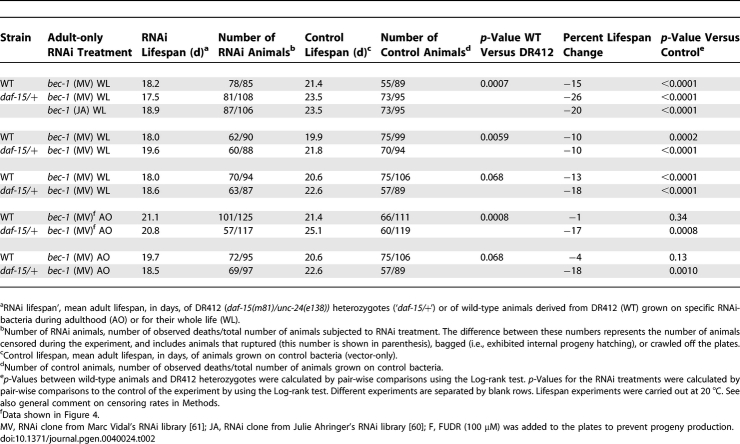
Lifespan Analysis of *daf-15* Heterozygotes Subjected to *bec-1* RNAi

We also investigated the level of autophagy in mutants heterozygous for the TOR-binding partner *daf-15*/Raptor [39]. We found that *daf-15* heterozygotes had increased levels of LGG-1::GFP-containing foci during development and as adults compared to wild-type animals (Figure 4B and data not shown). Thus, the TOR pathway appears to regulate autophagy in C. elegans.

To determine whether autophagy was likely to be required for the long lifespan of animals with reduced TOR activity, we asked whether the longevity of *daf-15*/Raptor mutants [39] (Table 2) required *bec-1*. We found that feeding bacteria expressing *bec-1* dsRNA to adult *daf-15* heterozygotes significantly shortened their lifespan in each of two independent experiments, but had no effect on wild-type animals (Figure 4C and 4D; Table 2). *bec-1* RNAi slightly shortened the lifespan of wild-type animals when administered throughout life (as reported earlier [22]), but we found that *bec-1* RNAi shortened the lifespan of the long-lived *daf-15* heterozygotes to a greater extent (Table 2). Taken together, these observations suggest that autophagy is required for the lifespan extension produced by the inhibition of TOR-pathway activity, and support the idea that dietary restriction induces autophagy via TOR inhibition in C. elegans.

### A Small GTPase, *rab-10*, Regulates Autophagy in Response to Dietary Restriction

The small GTPase *rab-10* appears to play a key role in the longevity response to dietary restriction in C. elegans [40]*.* As with TOR inhibition, *rab-10* inhibition extends the lifespan of normal, well-fed animals, but does not further extend the lifespan of animals subjected to dietary restriction. *rab-10* mRNA levels fall in response to dietary restriction, suggesting that the down-regulation of *rab-10* activity plays a causal role in the longevity response to dietary restriction. Like animals subjected to dietary restriction, animals with reduced *rab-10* activity also have delayed reproduction [40] and we found that they exhibited the dietary restriction-specific spectrofluorometric profile (Figure S4).

**Figure 5:**
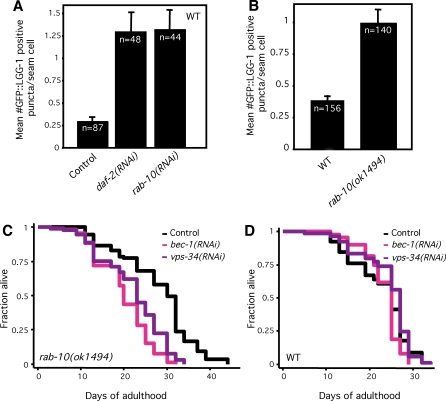
Inhibition of the Small GTPase *rab-10* Increases Autophagy (A) Average number of LGG-1::GFP-containing puncta in N2 wild-type animals (WT) fed either control bacteria or bacteria expressing *daf-2* (as a control) or *rab-10* dsRNA for two generations. *p* < 0.0001 for either *daf-2* or *rab-10* RNAi treatment compared to control RNAi treatment, unpaired, two-tailed t-test. *n*, total number of seam cells observed. Error bars: ±SEM. See Figure 1 for details. Please see Table S1 for quantification of all data. (B) Average number of LGG-1::GFP-containing puncta in *rab-10(ok1494)* mutants compared to N2 wild-type animals (WT), *p* < 0.0001, unpaired, two-tailed t-test. *n*, total number of seam cells observed. Error bars: ±SEM. Please see Figure 1 for details. Please see Table S1 for quantification of all data. (C) Survival curves of *rab-10(ok1494)* animals fed either control bacteria or bacteria expressing *bec-1* or *vps-34* dsRNA during adulthood at 20 °C. Mean lifespan was 27.9 d for control, 19.9 d for *bec-1* RNAi, and 22.1 d for *vps-34* RNAi*,* all pair-wise comparisons to control, *p* < 0.0001, Log-rank (Mantel-Cox) test. This experiment was performed two times. Please see Table 3 for additional data. (D) Survival curves of N2 wild-type animals (WT) fed either control bacteria or bacteria expressing *bec-1* or *vps-34* dsRNA during adulthood at 20 °C. These assays were performed at the same time as the *rab-10* lifespan analysis shown in Figure 5C. Mean lifespan was 22.9 d for control, 23.6 d for *bec-1* RNAi, and 24.5 d for *vps-34* RNAi, pair-wise comparison to control, *p* = 0.057 and *p* = 0.16, respectively, Log-rank (Mantel-Cox) test. Please see Table 1 for additional data.

RAB-10 is involved in vesicle transport in intestinal cells in C. elegans [41] and in mammalian epithelial cells [42,43]. In addition, RAB-10 was recently shown to regulate glutamate receptor recycling in neurons in C. elegans [44]. Because vesicle sorting is altered during autophagy, and because autophagy is increased in response to dietary restriction, we asked whether *rab-10* inhibition might trigger autophagy. To do this, we subjected wild-type animals carrying the LGG-1::GFP reporter to *rab-10* RNAi for their entire lives, and examined their L3 progeny. We found that this treatment, as well as the *rab-10(ok1494*) mutation, increased the number of LGG-1 foci in larvae (Figure 5A and 5B) and in adults (data not shown). We also asked if autophagy might be required for *rab-10* mutants to live long. To perform this experiment, we used a *rab-10(ok1494)* deletion mutant, which, as expected, was long-lived (Figure 5C and 5D; Table 3). We measured the lifespan of *rab-10(ok1494)* animals fed either *bec-1* or *vps-34* RNAi during adulthood, and we found that both RNAi clones significantly shortened lifespan (Figure 5C). Taken together, these findings suggest that *rab-10* inhibition is part of the mechanism by which dietary restriction stimulates autophagy.

**Figure 6:**
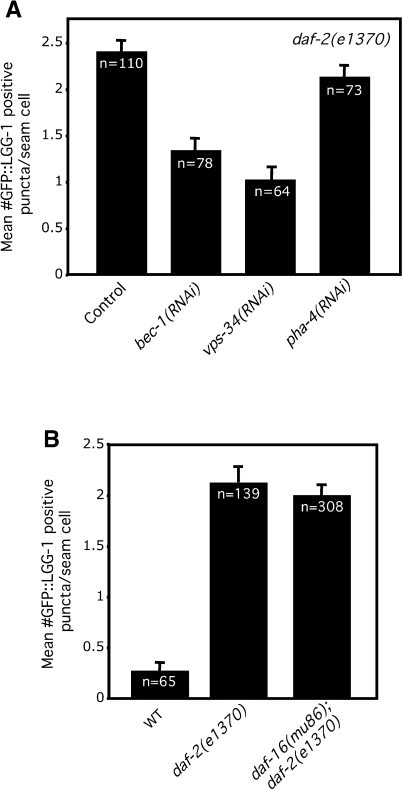
Increased Autophagy in *daf-2/*insulin/IGF Receptor Mutants Is Independent of the *daf-16*/FOXO and *pha-4*/FOXA Transcription Factors (A) Average number of LGG-1::GFP-containing puncta in *daf-2(e1370)* progeny of animals fed either control bacteria or bacteria expressing *bec-1*, *vps-34*, or *pha-4* dsRNA for their entire lives. *p* < 0.0001 for *bec-1* and *vps-34* RNAi-bacteria compared to control RNAi-bacteria, respectively, *p* = 0.17 for *pha-4* RNAi-bacteria compared to control RNAi-bacteria, unpaired, two-tailed t-test. *n*, total number of seam cells observed. Error bars: ±SEM. See Figure 1 for details. Feeding *daf-2* mutants for several generations with *pha-4* dsRNA sharply decreased the number of eggs laid (data not shown). The mean lifespan of *daf-2(e1370)* animals was shortened 12.5% by *pha-4* RNAi ([28] and data not shown), and we measured an 11% decrease in puncta in *daf-2(e1370)* animals fed *pha-4* RNAi. Even though this decrease was not statistically significant it remains possible that it relates to the small difference seen in lifespan. (B) Average number of LGG-1::GFP-containing puncta in *daf-16(mu86); daf-2(e1370)* double mutants compared to *daf-2(e1370)* animals; *p =* 0.50, unpaired, two-tailed t-test. N2 wild-type animals (WT) are shown for comparison. *n*, total number of seam cells observed. Error bars: ±SEM. See Figure 1 for details. The double mutant expressing the LGG-1 reporter had a mean lifespan similar to non-transgenic *daf-16*; *daf-2* double mutants (data not shown, [65]). Please see Table S1 for quantification of all data.

**Table 3:**
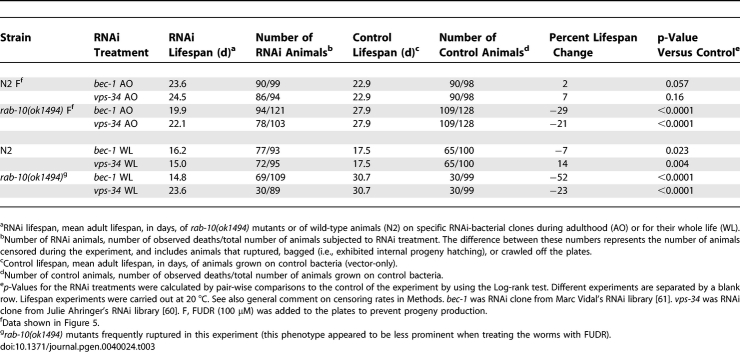
Lifespan Analysis of *rab-10(ok1494)* Mutants Subjected to *bec-1* or *vps-34* RNAi

### The FOXA Transcription Factor PHA-4 Is Required for Increased Autophagy in *eat-2* and *rab-10* Mutants

One could imagine that dietary restriction stimulates autophagy via purely post-translational mechanisms, such as changes in phosphorylation. However, recently the response to dietary restriction was shown to be subject to transcriptional regulation [28,45]. The FOXA transcription factor PHA-4 is required for the lifespan extension of animals subjected to dietary restriction [28]. Thus it was interesting to ask whether PHA-4 was required for dietary restriction to trigger autophagy. To do this, we fed *pha-4* RNAi-bacteria to *eat-2* animals expressing the LGG-1::GFP reporter for their entire lives and counted the number of GFP puncta in their progeny at the L3 stage. We found that the number of puncta was reduced significantly (Figure 3A, control bacteria: 1.00 ± 0.05 SEM, *pha-4* RNAi: 0.68 ± 0.04 SEM, *p* = 0.0001, unpaired t-test). Thus, changes in transcription mediated by PHA-4 are likely to be required for dietary restriction to trigger autophagy.

In principle, PHA-4 could trigger autophagy by reducing *rab-10* transcription in response to dietary restriction. In this model, *pha-4* would not be required to stimulate autophagy in animals already compromised for *rab-10* function. However, we found that feeding *pha-4* dsRNA significantly decreased the elevated number of LGG-1::GFP-containing foci in L3 stage *rab-10(ok1494)* mutants (Figure 3B). This finding suggests that *pha-4* acts either parallel to or downstream of *rab-10* to regulate autophagy.

### Increased Autophagy in *daf-2/*insulin/IGF Receptor Mutants Is Independent of the *daf-16*/FOXO Transcription Factor

Unlike animals subjected to dietary restriction, PHA-4/FOXA is not required for the increased longevity of *daf-2*/insulin/IGF-1-like mutants ([28] and confirmed by us [data not shown]). Consistent with this, we found that the elevated levels of LGG-1::GFP foci in L3 stage *daf-2(e1370)* mutants [22] were not significantly affected by subjecting the animals to *pha-4* RNAi (Figure 6A). The long lifespan of *daf-2* mutants is dependent on a different forkhead-family transcription factor, *daf-16/*FOXO [47]. Therefore, we asked whether *daf-16* was required for *daf-2* mutations to induce autophagy.

*daf-16* is known to act during adulthood to extend the lifespan of *daf-2* mutants [48]. We found that this was also the case for *bec-1* and *vps-34*: subjecting *daf-2(mu150)* animals to either *bec-1* or *vps-34* RNAi only during adulthood shortened lifespan (Figure S5; Table S2). This observation is consistent with earlier findings that treating *daf-2(e1370)* mutants with *bec-1* RNAi throughout their lives significantly shorten their lifespan [22]. To ask whether *daf-16* was required for autophagy in *daf-2* mutants, we introduced a *daf-16(null)* mutation into the *daf-2(e1370)* mutant and counted the number of LGG-1::GFP foci in the double mutant. We found that the *daf-16(mu86)* mutation had no effect on the level of foci in *daf-2(e1370)* larvae or adults (Figure 6B and data not shown). This finding suggests that *daf-16* is not required for the increased levels of autophagy in *daf-2* mutants, and, conversely, that autophagy is not sufficient to extend lifespan. In addition, we found that *bec-1* RNAi had no effect on the expression of any of the three transcriptional *daf-16* target genes we investigated (*sod-3*, *mtl-1* and *dod-8*, data not shown). Together, these findings suggest that *bec-1* and *daf-16* act in parallel pathways to increase the lifespan of *daf-2* mutants.

IThe great majority of the phenotypes observed in insulin/IGF-1-pathway mutants require the *daf-16/*FOXO transcription factor. Therefore, it was striking to find that autophagy appears to be induced in *daf-2* mutants independently of *daf-16*. While these studies were in progress, the Jacobson group showed that protein turnover in *daf-2* mutants is increased in a *daf-16*-independent fashion [49]; perhaps this turnover occurs, at least in part, via autophagy.

## Discussion

The process of autophagy allows an animal to recycle macromolecules during times of starvation and stress, presumably to deploy scarce resources in a more beneficial fashion. In this study, using a GFP reporter that indicates the presence of autophagic vesicles [22,32–35], we have shown that autophagy is triggered in long-lived animals subjected to dietary restriction in C. elegans. To test whether autophagy is required for the longevity of animals subjected to dietary restriction, we inhibited the activities of two genes required for autophagy, *bec-1* [*ATG6/VPS30/Beclin1*] and the PI 3-kinase *vps-34,* and found that the treatment prevented food-limited *eat-2* mutants from living long. Together, these findings suggest that autophagy is required for dietary restriction to extend lifespan. (We note that, while this paper was under revision, Beth Levine's group independently reported that autophagy genes are required for the longevity of *eat-2* mutants [34].) Disrupting genes required for autophagy did not perturb other phenotypes normally associated with dietary restriction, including morphological, spectrofluorimetric or reproductive changes. Thus, autophagy appears to be required specifically for lifespan extension. Perhaps autophagy allows an animal to clear away damaged proteins and other macromolecules that could accelerate the aging process and recycle their component amino acids into new cellular components.

Is it possible that this interpretation is incorrect, and that *bec-1* and *vps-34* actually have different functions in the animal that are required for longevity? In support of our interpretation, both *bec-1* and *vps-34* were required for the increased number of LGG-1::GFP-labeled autophagic vesicles we observed in *eat-2* and *daf-2* larvae (see Figures 3A and 6A) and LC3/LGG-1 is not known to have functions in processes other than autophagy. However, we did not observe changes in the adult LGG-1::GFP pattern when we produced changes in lifespan by inhibiting *bec-1* or *vps-1* function on day-1 of adulthood, though we were unable to assay LGG-1::GFP after day-2, when the adults are still very young (data not shown). This finding does not invalidate our interpretation, because it is possible that LGG-1::GFP recycling takes some time. Moreover, we observed the same phenomenon with two genes widely thought to influence autophagy: *daf-2* and *let-363/*TOR. (In our hands, *daf-2* and *let-363/*TOR RNAi administered on day-1 of adulthood lengthened lifespan but did not induce an autophagic phenotype by day-2 of adulthood [data not shown].)

What other functions could *bec-1* and *vps-34* have? In addition to their roles in autophagy, Vps34 is also required for endocytosis [50,51]. Likewise, *ATG6/VPS30/Beclin1* is involved in both autophagy and endocytosis in yeast, though Beclin1 is specifically involved in autophagy in mammals [52]. It is possible that *bec-1* also regulates endocytosis in *C. elegans,* although *C. elegans bec-1(+)* complements only the autophagy and not the vacuolar protein sorting function of yeast lacking VPS30 function [22]. Thus, knocking down *bec-1* and *vps-34* with RNAi could potentially shorten lifespan, at least in part, by blocking endocytosis. However, since *bec-1* and *vps-34* RNAi specifically affect the lifespans of long-lived mutants that have an elevated autophagic phenotype, we favor the interpretation that they shorten lifespan primarily by inhibiting autophagy.

In addition to recycling cytoplasmic contents, autophagy is also involved in non-apoptotic, programmed cell death [53]. Physiological levels of autophagy are essential to C. elegans cell survival during starvation, whereas excessive or insufficient levels of autophagy promote organismal death [35]. While it has not been observed so far, it is possible that non-autophagic cell death contributes to the longevity induced by dietary restriction.

We were prompted to investigate the role of autophagy in dietary restriction in part because autophagy is regulated by TOR, which in turn behaves as a downstream effector of the longevity response to dietary restriction in genetic tests [3,4,6]. In this study, we showed that TOR regulates autophagy in C. elegans and that genes required for autophagy are also required for the lifespan extension of TOR-pathway mutants. This finding suggests that autophagy is an integral part of the mechanism by which TOR inhibition increases lifespan, and supports the idea that dietary restriction extends lifespan via TOR inhibition.

**Figure 7:**
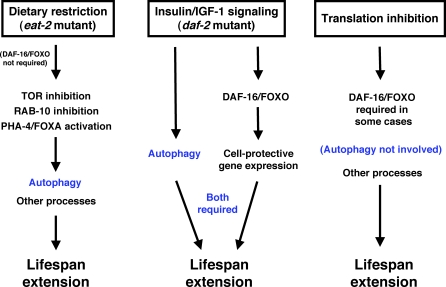
Model for the Role of Autophagy in Lifespan Extension by Dietary Restriction in C. elegans In response to dietary restriction using the *eat-2* mutation, TOR and RAB-10 activities fall, which triggers autophagy. Autophagy requires altered gene expression, since its appearance requires the transcription factor PHA-4. Autophagy is necessary but not sufficient for lifespan extension in long-lived *daf-2* insulin/IGF-1-receptor mutants; instead, autophagy and the DAF-16/FOXO transcription factor are both required, independently, for lifespan extension. Inhibiting protein synthesis in well-fed animals may activate a distinct longevity pathway, since inhibiting protein synthesis in the context of dietary restriction produces a lifespan extension that requires autophagy gene function, whereas inhibiting protein synthesis in well-fed animals produces a lifespan extension that is independent of autophagy gene function. The *sir-2* histone deacetylase is not in this diagram, as we [6] and several other groups [66–68] have found that *sir-2* deletion mutations do not prevent dietary restriction from increasing lifespan in *C. elegans.*

TOR inhibition also reduces the rate of protein synthesis, and inhibiting protein synthesis is sufficient to extend lifespan. Previously we suggested that the longevity of TOR mutants might be caused, in part, by reduced protein synthesis [6]. However, these and other new findings put a new twist into this line of reasoning. Recently, Kapahi's group showed that *bec-1* RNAi does not prevent S6-kinase/*rsks-1* or eIF-4G/*ifg-1* mutations, which reduce protein synthesis, from extending lifespan in C. elegans [9]. We observed this, as well, for *rsks-1(sv31)* and *ife-2(ok306)* (Figure S6A; Table S3; and data not shown). In addition, we looked for LGG-1::GFP foci in *rsks-1* mutants in which protein synthesis had been inhibited and failed to see any increase in the number of LGG-1::GFP positive foci in seam cells (Figure S6B). While it is possible that autophagy is taking place in other cells/tissues in the animal, the simplest interpretation of these findings is that the lifespan extension produced by the inhibition of protein synthesis does not involve autophagy. Thus, these findings raise an interesting question: If protein synthesis falls in response to dietary restriction, and the lifespan extension produced by inhibiting protein synthesis does not involve autophagy, why is the lifespan extension produced by dietary restriction dependent on autophagy genes? One possibility is that disrupting protein synthesis in well-fed animals triggers a novel, lifespan-extending pathway that is not triggered by dietary restriction (see model in Figure 7). This seems plausible, since the reduction in protein synthesis caused by dietary restriction takes place in the context of a global physiological shift that down-regulates many other growth-related processes. Consistent with the idea that dietary restriction/TOR inhibition and direct protein synthesis activate distinct longevity pathways, the lifespan of *eat-2* mutants is further extended by direct protein synthesis inhibition but not by TOR inhibition [6]. The idea that inhibiting protein synthesis in well-fed animals activates a novel longevity pathway does not rule out the possibility that the decrease in protein synthesis that occurs in response to TOR inhibition or dietary restriction, like autophagy, is required for increased longevity. It will be interesting to explore these pathways in more detail with biochemical and molecular experiments.

Our studies have placed two new genes into the pathway by which dietary restriction triggers autophagy, *rab-10* and *pha-4. rab-10* encodes a small GTPase whose mRNA levels fall in response to dietary restriction. *rab-10* inhibition appears to be part of the mechanism by which dietary restriction extends lifespan. When *rab-10* is inhibited with RNAi, a robust dietary-restriction phenotype (lifespan extension, delayed reproduction, spectroflurorimetric shift) is produced ([40] and Figure S4), and *rab-10* RNAi does not further extend the lifespan of *eat-2* mutants [40]. Our findings indicate that *rab-10* inhibition stimulates autophagy. RAB-10 is involved in vesicle transport in intestinal cells in C. elegans [41] and in mammalian epithelial cells [42,43]. RAB-10 has also been shown to regulate glutamate receptor recycling in neurons in C. elegans [44]. Together these findings suggest the hypothesis that dietary restriction alters patterns of vesicle transport in a way that triggers autophagy and perhaps other events that promote lifespan extension.

The transcription factor PHA-4, which is required for the longevity of animals subjected to dietary restriction, is required for the elevated number of autophagic vesicles observed in *eat-2* mutants [28]. Thus, the increase in autophagy that occurs in response to dietary restriction is not a passive consequence of food limitation but is likely to require changes in gene expression. It will be interesting to learn what genes act downstream of *pha-4* to regulate autophagy. PHA-4 is also required for the inhibition of *rab-10* to induce autophagy, so *pha-4* may act downstream of *rab-10* in the autophagy pathway. Perhaps changes in the pattern of vesicle transport are part of the signal that activates PHA-4 in response to dietary restriction. Alternatively, in food-limited animals, PHA-4 could regulate the expression of one or more genes that acts in the context of altered vesicle metabolism to induce autophagy.

Autophagy seems like such a “purifying” process that it is tempting to think that it might be sufficient to extend lifespan. However, our findings suggest that is not the case. The longevity of *daf-2* insulin/IGF-1 receptor mutants requires the FOXO-family transcription factor DAF-16. Surprisingly, we found that *daf-16(null); daf-2(-)* double mutants had the same high level and distribution of autophagic LGG-1::GFP puncta as did *daf-2(-)* single mutants. The fact that *daf-16; daf-2* double mutants are not long-lived ([47] and data not shown) suggests that autophagy is not sufficient to increase lifespan.

Why are transcription factors as well as autophagy required for lifespan extension in *daf-2* mutants? DAF-16/FOXO is known to stimulate the expression of a wide variety of antioxidant, chaperone, antimicrobial, metabolic and other genes that act in a cumulative fashion to extend lifespan [54–57]. Perhaps the role of autophagy in the longevity of *daf-2* mutants is to provide new raw material for protein construction by recycling damaged cellular components, and the role of DAF-16 is to channel this raw material into proteins that protect and repair cells, and thereby extend lifespan.

Not only is autophagy insufficient to extend lifespan, it is not necessary for lifespan extension. We found that subjecting the long-lived mitochondrial mutants *clk-1* and *isp-1* to *bec-1* or *vps-34* RNAi during adulthood has no effect on lifespan (Figure S7; Table S3) (though autophagy could conceivably play a longevity role in mitochondrial respiration during development; Table S4). In addition, as discussed above, inhibiting protein synthesis in otherwise well-fed animals extends lifespan in the absence of autophagy. Taken together, these findings suggest that autophagy may be required specifically for longevity pathways that are fully integrated with, and regulated by, environmental signals that reflect the availability of food, such as the insulin/IGF-1 pathway and the response to dietary restriction.

## Methods

### Strains.

All strains were maintained as previously described [58]. Single mutants: CF1037: *daf-16(mu86) I*, CF2846: *rab-10(ok1494) I* (VC1026 outcrossed four times to Kenyon lab N2 wild-type strain), CF1908: *eat-2(ad1116) II* (DA1116 outcrossed four times to Kenyon lab N2 wild-type strain), CF1041: *daf-2(e1370) III*, CF512: *fer-15(b26) II; fem-1(hc17) III*. CF1844: *fer-15(b26) II; daf-2(mu150) III; fem-1(hc17) IV*. VB633: *rsks-1(sv31) III*. Double mutants: CF1850: *eat-2(ad1116) rrf-3(pk1426) II*, CF2120: *daf-2(mu150) III; ced-3(n1289) IV*, CF2140: *eat-2(ad1116) II; ced-3(n1289) IV* [59], DR412: *daf-15(m81)/unc-24(e138) IV* [39]. Transgenic strains: QU1: izEx1[*Plgg-1::gfp::lgg-1* + *rol-6*] ([22], named in this study), QU2: *daf-2(e1370)*; izEx1[*Plgg-1::gfp::lgg-1* + *rol-6*] [22], named in this study), CF2494: *eat-2(ad1116);* izEx1[*Plgg-1::gfp::lgg-1* + *rol-6*], CF2946: *eat-2(ad1116) rrf-3(pk1426);* izEx1[*Plgg-1::gfp::lgg-1* + *rol-6*], CF2544: *daf-16(mu86); daf-2(e1370);* izEx1[*Plgg-1::gfp::lgg-1* + *rol-6*], CF2821: *daf-15(m81)/unc-24(e138);* izEx1[*Plgg-1::gfp::lgg-1* + *rol-6*], CF2864: *rab-10(ok1494);* izEx1[*Plgg-1::gfp::lgg-1* + *rol-6*], CF2865: *rsks-1(sv31);* izEx1[*Plgg-1::gfp::lgg-1* + *rol-6*], CF2866: *isp-1(qm150);* izEx1[*Plgg-1::gfp::lgg-1* + *rol-6*].

### RNAi clone analysis.

The identity of all RNAi clones was verified by sequencing the inserts using the M13-forward primer. The TOR RNAi clone was obtained from Dr. Xiaomeng Long, Massachusetts General Hospital. The *daf-2* RNAi clone was published previously [48]. All other clones were from Julie Ahringer's RNAi library [60] or Marc Vidal's RNAi library [61].

The gene *bec-1* is part of an operon that contains the stress-inducible transcription factor gene *skn-1*, which is required for the lifespan extension induced by dietary restriction [45]. Non-specific inactivation of genes in operons by RNAi has been observed [62,63]. However, using quantitative RT-PCR, we found that RNAi of *bec-1* did not affect the mRNA levels of *skn-1* (data not shown). Thus, the phenotypes observed in animals treated with *bec-1* RNAi are likely to originate from reduced *bec-1* mRNA levels.

### Lifespan analysis.

Lifespan analysis was conducted at 20 °C as described previously [40] unless stated otherwise. RNAi treatments were either performed as whole-life treatments or adult-only treatments. In the whole-life RNAi treatments, eggs were added to plates seeded with the RNAi-bacteria of interest. In the adult-only analysis, eggs were added to plates seeded with RNAi vector-only bacteria, and adult animals were transferred to gene-specific RNAi-bacterial plates. The chemical 2'fluoro-5′deoxyuridine (FUDR, Sigma) was sometimes added to adult worms (100 μM) to prevent their progeny from developing. During this project, we experienced a time period in which the *bec-1* RNAi clone failed to shorten the lifespan of *eat-2* mutants in the presence of FUDR. These experiments were not included in this publication and we continued our experiments without FUDR. At least 80 worms were tested in each experiment. Strains were grown at 20 °C under optimal growth conditions for at least two generations before use in lifespan analysis. During the analysis of large numbers of RNAi clones, CF512 or N2 controls were performed either concurrently or in overlapping time frames. In all experiments, the pre-fertile period of adulthood was used as *t* = 0 for lifespan analysis. Censoring in the lifespan analysis included animals that ruptured, bagged (i.e., exhibited internal progeny hatching), or crawled off the plates. STATA software was used for statistical analysis and to determine means and percentiles. In all cases, *p* values were calculated using the Log-rank (Mantel-Cox) method.

### Analysis of autophagic events using an LGG-1 reporter strain.

The level of autophagy in various mutants was assessed using an LGG-1::GFP translational reporter characterized previously [22]. Animals were raised at 20 °C. GFP-positive puncta were counted (using 1000-fold magnification on a Zeiss Axioplan II microscope) in the seam (lateral epidermal) cells of L3 transgenic animals, which were staged by gonad morphology and germline developmental phenotype. Counting puncta during adulthood was difficult due to the increased level of endogenous autofluorescence in the animal (data not shown). In addition, examining puncta in adults was complicated by the difficulty in identifying seam cells. Between 3–10 seam cells were examined in each of 10–40 animals from at least two independent trials and averaged (see Table S1). Data analysis was done using unpaired, two-tailed t-test. When performing RNAi experiments to count LGG-1::GFP-positive foci, young adults were fed the RNAi bacteria, and the L3 progeny of their progeny (“F_2_ generation”) were examined. Analyzing the L3 animals in the first generation, even in *daf-2* positive controls, was not sufficient to change the number of foci by the L3 stage (data not shown).

GFP-positive punctate areas were also counted in wild-type animals (QU1) subjected to dietary restriction by direct food limitation. The bacterial culture was grown in a slightly modified, scaled up version of the protocol described in Gerstbrein *et al.* [31], to yield cultures corresponding to ad libitum (AL, or fully fed) and dietary-restricted (DR, or food-limited) conditions. 500 μl of E. coli OP50 (OD600 ∼1) was inoculated into 250 ml LB, grown for 5 hrs at 37 °C and resuspended in 25 ml complete S-basal medium. This culture corresponded to the stock as well as the ‘AL' culture. Cell density of the stock was determined by counting DAPI stained cells in a Petroff-Hausser counting chamber. The ‘AL' culture corresponded to a cell density of 1.9 × 10^10^ cells/ml and was diluted in complete S-basal medium to yield the ‘DR' culture (2.6 × 10^9^ cells/ml). Worms grown in more dilute culture of cell density 5.2 × 10^8^ cells/ml appeared to border on starvation while the worms grown in culture of cell density 2.6 × 10^8^ cells/ml arrested. About 25 eggs were added to wells of a 24-well plate containing 600 μl of the bacteria-supplemented S-basal medium each and cultured at 20 °C with shaking. The media was changed every other day once the eggs developed into adults. Worms grown in the ‘DR' culture were considered to be dietary-restricted as they developed with a slight lag as compared to animals in ‘AL' culture and they had lower AGE pigments (a biomarker of better healthspan and lifespan, data not shown). L3 animals were observed after ∼53 hours in ‘AL' culture and after ∼60 hours in ‘DR' culture. GFP-positive foci were counted in hypodermal seam cells of L3 transgenic animals.

We note that although the genetic requirements for the longevity of *eat-2* mutants and animals subjected to dietary restriction in liquid media are similar to one another [26,29,30], initiating dietary restriction in a third way; namely, on plates during mid-adulthood, produces a lifespan increase with at least some different genetic requirements [64]. Therefore, it is possible that the role and regulation of autophagy in animals subjected to dietary restriction in different ways may not be the same.

### Brood-size assay.

Eggs were incubated at 20 °C on control plates and 16 late-L4 stage worms were picked for each treatment and transferred to fresh RNAi or OP50 plates every 12 hours for 4–5 days. After this period, the worms were transferred every 24 hours. Worms that crawled off the plates, bagged or ruptured were censored. All progeny plates were incubated at 20 °C for about 2 days following transfer of the parental worms and then held at 4 °C. The number of worms that developed was determined at the end of the experiment.

### Fluorescence spectroscopy.

In vivo autofluorescence in C. elegans was measured using a spectrofluorimeter (Fluorolog®-3, Jobin Yvon Inc., Edison NJ) equipped with a plate reader (MicroMax 384). For each time point/scan, 50 animals per RNAi clone were cleaned on unseeded NGM plates, and then transferred to 50 μl of 10 mM NaN_3_ in a single well of a 96-well plate (Cat #437842, Nalge Nunc Internat'l). TRP and AGE fluorescence intensities and the excitation wavelength for maximal AGE fluorescence intensity were measured as described [31]. Each scan was done in triplicate. Data analysis was done using unpaired, one-tailed t-test.

## Supporting Information

Figure S1 Pumping Rates of *eat-2(ad1116)* Mutants and Wild-type (N2) Animals Grown on *bec-1* or *vps-34* RNAi-bacteria from Hatching. (48 KB PPT)

Figure S2 Inhibition of *bec-1* from the Time of Hatching Does Not Change the Progeny Profile of *eat-2(ad1116)* Mutants (67 KB PPT)

Figure S3 Inhibition of *bec-1* in *eat-2(ad1116)* Mutant Adults Does Not Significantly Alter Their Dietary-restriction Fluorimetric Profile (96 KB PPT)

Figure S4 Inhibition of *rab-10* Triggers a Fluorimetric Dietary Restriction Signature in Wild-type Animals (84 KB PPT)

Figure S5 Inhibition of *bec-1* and *vps-34* during Adulthood Shortens the Long Lifespan of *daf-2* Mutants (54 KB PPT)

Figure S6 Animals with Reduced Translation Do Not Require *bec-1* Gene Activity during Adulthood to Live Long (56 KB PPT)

Figure S7 Animals with Reduced Mitochondrial Respiration Do Not Require *bec-1* during Adulthood to Live Long (83 KB PPT)

Table S1 Analysis of Strains Expressing the Transgene LGG-1::GFP (99 KB DOC)

Table S2 Lifespan Analysis of *daf-2(mu150)* Mutants Subjected to *bec-1* or *vps-34* RNAi during Adulthood (71 KB DOC)

Table S3 Lifespan Analysis of *rsks-1*, *isp-1*, and *clk-1* Mutants Subjected to *bec-1* RNAi during Adulthood (65 KB DOC)

Table S4 Lifespan Analysis of Strains Grown on RNAi Clones for Autophagy-associated Genes *bec-1* and *vps-34* throughout Their Whole Life. (63 KB DOC)
