# Supplementary material for: A Role for Autophagy in the Extension of Lifespan by Dietary Restriction in C. elegans
Source: PLoS Genet. 2008 Feb 15;4(2):e24. doi: 10.1371/journal.pgen.0040024 (PMC2242811; doi:10.1371/journal.pgen.0040024)
Supplement: Supplementary file 1 [file pgen.0040024.st001.doc]

Table S1

| **Data from Fig. #** | **Strain** | **LGG-1::GFP expressing strain** | **RNAi treatment** | **Average # puncta/ seam cell** | **SEM** | **N_seam cells_** | **N_animals_** | ***P* value** |
| --- | --- | --- | --- | --- | --- | --- | --- | --- |
| 1B | QU1 | N2 | - | 0.36 | 0.05 | 216 | 28 |  |
|  | CF2544 | *eat-2(ad1116)* | - | 0.80 | 0.07 | 266 | 45 | <0.0001 |
| 1C | QU1 | N2, normal food | - | 0.21 | 0.04 | 126 | 20 |  |
|  | QU1 | N2, dietary restricted | - | 1.3 | 0.17 | 116 | 19 | <0.0001 |
| 3A | CF2494 | *eat-2(ad1116)* | Control | 1.00 | 0.05 | 217 | 44 |  |
|  |  |  | *bec-1* | 0.64 | 0.05 | 186 | 29 | <0.0001 |
|  |  |  | *vps-34* | 0.50 | 0.07 | 106 | 19 | <0.0001 |
|  |  |  | *pha-4* | 0.68 | 0.04 | 387 | 56 | 0.0001 |
| 3B | CF2864 | *rab-10(ok1494)* | Control | 1.07 | 0.13 | 29 | 11 |  |
|  |  |  | *pha-4* | 0.36 | 0.06 | 90 | 21 | <0.0001 |
| 4A | QU1 | N2 | Control | 0.28 | 0.03 | 96 | 14 |  |
|  |  |  | *let-363* | 0.88 | 0.11 | 92 | 22 | <0.0001 |
| 4B | QU1 | N2 | - | 0.33 | 0.05 | 171 | 24 |  |
|  | CF2821 | *daf-15/+* | - | 0.74 | 0.06 | 256 | 45 | <0.0001 |
| 5A | QU1 | N2 | Control | 0.29 | 0.06 | 87 | 25 |  |
|  |  |  | *daf-2* | 1.29 | 0.22 | 48 | 12 | <0.0001 |
|  |  |  | *rab-10* | 1.31 | 0.22 | 44 | 13 | <0.0001 |
| 5B | QU1 | N2 | - | 0.38 | 0.05 | 156 | 25 |  |
|  | CF2864 | *rab-10(ok1494)* | - | 0.99 | 0.11 | 140 | 27 | <0.0001 |
| 6A | QU2 | *daf-2(e1370)* | Control | 2.41 | 0.13 | 110 | 24 |  |
|  |  |  | *bec-1* | 1.35 | 0.14 | 78 | 16 | <0.0001 |
|  |  |  | *vps-34* | 1.03 | 0.15 | 64 | 11 | <0.0001 |
|  |  |  | *pha-4* | 2.14 | 0.15 | 73 | 12 | 0.17 |
| 6B | QU1 | N2 | - | 0.28 | 0.07 | 65 | 10 |  |
|  | QU2 | *daf-2(e1370)* | - | 2.13 | 0.15 | 139 | 32 |  |
|  | CF2544 | *daf-16(mu86); daf-2(e1370)* | - | 2.00 | 0.10 | 308 | 59 | 0.50 |
| S6B | QU1 | N2 | - | 0.49 | 0.07 | 103 | 17 |  |
|  | CF2865 | *rsks-1(sv31)* | - | 0.46 | 0.06 | 157 | 24 | 0.87 |
| S7B | QU1 | N2 | - | 0.39 | 0.06 | 120 | 17 |  |
|  | CF2866 | *isp-1(qm150)* | - | 0.65 | 0.05 | 317 | 37 | 0.0026 |
| S7C | QU1 | N2 | - | 0.42 | 0.05 | 162 | 23 |  |
|  | CF2863 | *clk-1(qm30)* | - | 0.57 | 0.05 | 223 | 31 | 0.038 |

**Table S1. Analysis of strains expressing the transgene LGG-1::GFP.**

Summary of LGG-1::GFP positive puncta in L3 larvae of different genetic backgrounds using the LGG-1::GFP reporter described in [22]. The average number of puncta per seam cell was calculated from at least two independent trials. N_seam cells_, number of seam cells analyzed, N_animals_, number of animals in which seam cells were analyzed. *P* values were calculated as unpaired, two-tailed *t*-test. Animals were raised at 20º.
