# Supplementary material for: A Role for Autophagy in the Extension of Lifespan by Dietary Restriction in C. elegans
Source: PLoS Genet. 2008 Feb 15;4(2):e24. doi: 10.1371/journal.pgen.0040024 (PMC2242811; doi:10.1371/journal.pgen.0040024)
Supplement: Supplementary file 2 [file pgen.0040024.st002.doc]

**Table S2**

| **Strain** | **Adult-only RNAi treatment** | **RNAi**  **lifespan (days)** | **Number of RNAi animals** | **Control lifespan (days)** | **Number of control animals** | **% lifespan change** | ***P* value *versus* control** |
| --- | --- | --- | --- | --- | --- | --- | --- |
| N2 | *bec-1* (MV) | 18.9 | 40/91 | 17.5 | 58/87 | 8 | 0.045 |
|  | *bec-1* (MV) | 19.0 | 84/111 | 19.7 | 79/115 | -4 | 0.14 |
|  | *bec-1 (*MV) | 18.5 | 67/91 | 19.5 | 79/89 | -5 | 0.17 |
|  | *bec-1 (*MV) | 19.8 | 68/92 | 19.8 | 70/86 | 0 | 0.65 |
| CF1844 | *daf-16* | 16.8 | 87/98 | 33.6 | 90/95 | -50 | <0.0001 |
| *daf-2(mu150)* | *daf-16** | 20.2 | 77/100 | 30.9 | 62/99 | -35 | <0.0001 |
|  | *daf-16* | 18.9 | 104/107 | 34.1 | 101/107 | -45 | <0.0001 |
|  | *daf-16* | 16.6 | 96/100 | 32.7 | 50/98 | -49 | <0.0001 |
|  | *bec-1* (MV) | 25.4 | 82/85 | 28.4 | 71/75 | -11 | 0.022 |
|  | *bec-1* (MV) | 27.4 | 87/97 | 31.0 | 63/87 | -12 | 0.0002 |
|  | *bec-1* (MV) | 26.8 | 84/91 | 34.1 | 101/107 | -21 | <0.0001 |
|  | *bec-1* (MV) | 24.0 | 90/100 | 32.7 | 50/98 | -27 | <0.0001 |
|  | *bec-1* (MV)* | 21.3 | 62/101 | 30.9 | 62/99 | -31 | <0.0001 |
|  | *bec-1* (MV) | 22.4 | 86/106 | 33.6 | 90/95 | -33 | <0.0001 |
|  | *vps-34* (JA) | 26.4 | 82/86 | 28.4 | 71/75 | -7 | 0.025 |
|  | *vps-34* (MV) | 24.9 | 77/81 | 28.4 | 71.75 | -12 | 0.0001 |
|  | *vps-34* (JA)* | 24.0 | 70/91 | 30.9 | 62/99 | -22 | <0.0001 |

**Table S2. Lifespan analysis of *daf-2(mu150)* mutants subjected to *bec-1* or *vps-34* during adulthood.**

‘RNAi lifespan’, mean adult lifespan, in days, of sterile *fer-15(b26); daf-2(mu150); fem-1(hc17)* mutants (CF1844) on *bec-1* RNAi-bacterial clones during adulthood. ‘Number of RNAi animals’, number of observed deaths/total number of animals subjected to RNAi treatment. The difference between these numbers represents the number of animals censored during the experiment, and includes animals that ruptured, bagged (ie, exhibited internal progeny hatching) or crawled off the plates. ‘Control lifespan’, mean adult lifespan, in days, of animals grown on control bacteria (vector-only). ‘Number of control animals’, number of observed deaths/total number of animals grown on control bacteria. *p* values were calculated by pair-wise comparisons to the control of the experiment by using the Log-rank test. Repetitions of the same experiments are listed in order. Lifespan experiments were carried out at 20°C. See also general comment on censoring rates in Experimental procedures. ‘MV’, RNAi clone from Marc Vidal’s RNAi library [61]. ‘JA’, RNAi clone from Julie Ahringer’s RNAi library [60]. ‘*’, data shown in Fig. S5.
