# Supplementary material for: A Role for Autophagy in the Extension of Lifespan by Dietary Restriction in C. elegans
Source: PLoS Genet. 2008 Feb 15;4(2):e24. doi: 10.1371/journal.pgen.0040024 (PMC2242811; doi:10.1371/journal.pgen.0040024)
Supplement: Supplementary file 3 [file pgen.0040024.st003.doc]

**Table S3**

| **Strain** | **Adult-only RNAi treatment** | **RNAi**  **lifespan (days)** | **Number of RNAi animals** | **Control lifespan (days)** | **Number of control animals** | **% lifespan change** | ***P* value *versus* control** |
| --- | --- | --- | --- | --- | --- | --- | --- |
| VB633 | *bec-1* (MV) | 24.6 | 61/95 | 21.8 | 59/95 | 13 | 0.0001 |
| *rsks-1(sv31)* | *bec-1* (MV)* | 23.4 | 24/62 | 22.1 | 42/72 | 6 | 0.23 |
|  | *bec-1* (MV) F | 24.8 | 56/70 | 22.2 | 67/91 | 12 | 0.029 |
|  | *bec-1* (JA) F | 22.1 | 54/75 | 22.2 | 67/91 | 0 | 0.80 |
| CF2354 | *bec-1* (MV) | 18.7 | 54/105 | 21.4 | 78/108 | -13 | 0.017 |
| *isp-1(qm150)* | *bec-1* (MV) F | 23.7 | 65/115 | 25.2 | 58/115 | -6 | 0.076 |
|  | *bec-1* (MV) F | 25.1 | 78/116 | 26.0 | 68/114 | -3 | 0.27 |
|  | *bec-1* (MV)* | 21.9 | 64/89 | 23.1 | 55/72 | -5 | 0.15 |
|  | *bec-1* (JA) F | 24.5 | 67/118 | 25.2 | 58/115 | -3 | 0.30 |
|  | *bec-1* (JA) F | 24.4 | 73/121 | 26.0 | 68/114 | -6 | 0.022 |
| CF2172 | *bec-1* (MV) F | 25.6 | 72/92 | 23.9 | 73/96 | 7 | 0.071 |
| *clk-1(qm30)* | *bec-1* (MV) *F | 24.7 | 70/90 | 24.2 | 73/95 | 2 | 0.84 |
|  | *bec-1* (MV) F | 23.5 | 73/95 | 24.2 | 73/95 | -3 | 0.20 |
|  | *bec-1* (MV) F | 22.5 | 69/91 | 23.9 | 73/96 | -6 | 0.019 |

**Table S3. Lifespan analysis of *rsks-1, isp-1* and *clk-1* mutants subjected to *bec-1* RNAi during adulthood.**

‘RNAi lifespan’, mean adult lifespan, in days, of *rsks-1(sv31), isp-1(qm150)* *or clk-1(qm30)* mutants on *bec-1* RNAi-bacterial clones during adulthood. ‘Number of RNAi animals’, number of observed deaths/total number of animals subjected to RNAi treatment. The difference between these numbers represents the number of animals censored during the experiment, and includes animals that ruptured, bagged (ie, exhibited internal progeny hatching) or crawled off the plates. ‘Control lifespan’, mean adult lifespan, in days, of animals grown on control bacteria (vector-only). ‘Number of control animals’, number of observed deaths/total number of animals grown on control bacteria. *P* values were calculated by pair-wise comparisons to the control of the experiment by using the Log-rank test. Repetitions of the same experiments are listed in order. Lifespan experiments were carried out at 20°C. See also general comment on censoring rates in Experimental procedures. ‘MV’, RNAi clone from Marc Vidal’s RNAi library [61]. ‘JA’, RNAi clone from Julie Ahringer’s RNAi library [60]. ‘F’, FUDR (100 μM) was added to the plates to prevent progeny production. ‘*’, data shown in Fig. S6A, S7A and S7C.

We confirmed that adult-only RNAi works equally well in adult *rsks-1* mutants and wild-type animals by quantitative RT-PCR analysis [69] of animals reared on *bec-1* RNAi-bacteria from L4 to D2 of adulthood. In these experiments (two repeats), *bec-1* RNAi reduced *bec-1* mRNA levels ~3-4 fold in both *rsks-1* mutants and in wild-type animals (S. Taubert, unpublished data). It is possible that *isp-1* and *clk-1* mutants do not respond to adult-only *bec-1* RNAi treatment, though this seems unlikely since at least *isp-1* mutants do respond to other adult-only RNAi treatments [6].
