# Supplementary material for: A Role for Autophagy in the Extension of Lifespan by Dietary Restriction in C. elegans
Source: PLoS Genet. 2008 Feb 15;4(2):e24. doi: 10.1371/journal.pgen.0040024 (PMC2242811; doi:10.1371/journal.pgen.0040024)
Supplement: Supplementary file 4 [file pgen.0040024.st004.doc]

**Table S4**

| **Strain** | **Whole-life RNAi treatment** | **RNAi**  **lifespan (days)** | **Number of RNAi animals** | **Control lifespan (days)** | **Number of control animals** | **% lifespan change** | ***P* value *versus* control** |
| --- | --- | --- | --- | --- | --- | --- | --- |
| N2 | *bec-1* (MV) | 16.2 | 77/93 | 17.5 | 65/100 | -7 | 0.023 |
|  | *bec-1* (MV) | 14.5 | 69/96 | 19.8 | 70/86 | -27 | <0.0001 |
|  | *vps-34* (JA) | 15.0 | 72/95 | 17.5 | 65/100 | -14 | 0.0004 |
| CF1908 | *bec-1* (MV) | 14.1 | 57/81 | 22.4 | 46/77 | -37 | <0.0001 |
| *eat-2(ad1116)* | *vps-34* (JA) | 15.5 | 54/85 | 22.4 | 46/77 | -31 | <0.0001 |
| CF1850  *eat-2; rrf-3* | *bec-1* (JA) | 14.9 | 76/91 | 19.3 | 70/92 | -23 | <0.0001 |
| CF1844 | *daf-16* | 18.2 | 96/101 | 30.9 | 62/99 | -41 | <0.0001 |
| *daf-2(mu150)* | *bec-1* (MV) | 22.6 | 88/102 | 30.9 | 62/99 | -27 | <0.0001 |
|  | *vps-34* (JA) | 22.8 | 91/107 | 30.9 | 62/99 | -41 | <0.0001 |
| CF2172 | *bec-1* (MV) | 17.1 | 81/111 | 19.5 | 87/110 | -12 | 0.013 |
| *isp-1(qm150)* | *bec-1* (JA) | 17.6 | 83/103 | 19.5 | 87/110 | -10 | 0.056 |
|  | *bec-1* (MV) | 13.5 | 60/92 | 23.1 | 55/72 | -42 | <0.0001 |

**Table S4. Lifespan analysis of strains grown on RNAi clones for autophagy-associated genes *bec-1* and *vps-34* throughout their whole life.**

‘RNAi lifespan’, mean adult lifespan, in days, of various strains on different RNAi-bacterial clones during adulthood. ‘Number of RNAi animals’, number of observed deaths/total number of animals subjected to RNAi treatment. The difference between these numbers represents the number of animals censored during the experiment, and includes animals that ruptured, bagged (ie, exhibited internal progeny hatching) or crawled off the plates. ‘Control lifespan’, mean adult lifespan, in days, of animals grown on control bacteria (vector-only). ‘Number of control animals’, number of observed deaths/total number of animals grown on control bacteria. *P* values were calculated by pair-wise comparisons to the control of the experiment by using the Log-rank test. Repetitions of the same experiments are listed in order. Lifespan experiments were carried out at 20°C. See also general comment on censoring rates in Experimental procedures. ‘MV’, RNAi clone from Marc Vidal’s RNAi library [61]. ‘JA’, RNAi clone from Julie Ahringer’s RNAi library [60].

We note that whole-life treatments with *bec-1* RNAi in general had significant lifespan-shortening effects on all the different long-lived *C. elegans* mutants we tested, along with wild-type animals. Since *bec-1* is essential for normal development [22, 37], we therefore cannot exclude the possibility that inhibition of *bec-1* during this period limits a general factor that is vital for the animal*.* However, it is possible that the lifespan extending mechanism induced by *isp-1* mutants is set during development [during this period, we find *isp-1* mutants to have slightly increased levels of autophagy, Fig. S7B – a similar trend might be true for *clk-1* mutants. Fig. S7D] and this mechanism requires autophagy.
